# Cytomegalovirus Retinitis in a Patient on Long-term Mycophenolate Mofetil Treatment for Myasthenia Gravis

**DOI:** 10.18502/jovr.v15i3.7463

**Published:** 2020-07-29

**Authors:** Shyam Patel, Alexander Robin

**Affiliations:** ^1^Department of Ophthalmology, Cook County Hospital, Chicago, IL, USA

##  PRESENTATION

**Figure 1:**
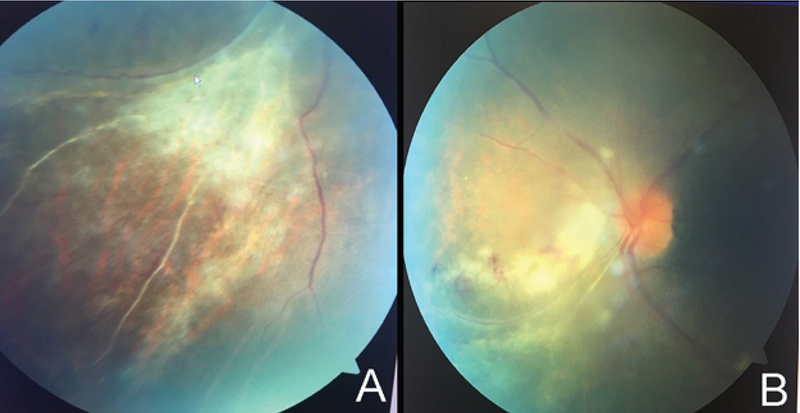
(A) A funduscopic photo of the left eye shows fluffy white lesions with intraretinal hemorrhage predominantly in the inferonasal quadrant. (B) A funduscopic photo of the left inferonasal quadrant five weeks after treatment initiation.

A 64-year-old man with myasthenia gravis (MG) presented with blurry vision in his left eye (OS). His visual acuity was 20/20 in the right eye and 20/50 OS. His intraocular pressures, pupils, and anterior segment were normal. He had 1+ vitritis and vaso-occlusive appearance of the retina with sclerotic vessels and hemorrhages in the inferonasal quadrant OS (Figure 1), consistent with features of cytomegalovirus (CMV) retinitis. He was immunocompromised secondary to mycophenolate mofetil (MMF) administered for MG, with 0.6% lymphocytes (normal: 26.0–46.0%), a lymphocyte count of 0.1×10${}^{3}$ cells/μL, and a leukocyte count of 11.3×10${}^{3}$ cells/μL. He was receiving 1000 mg of MMF BID. The human immunodeficiency virus (HIV) test result was negative, and no further workup for immunosuppression, including cancer, was conducted.

**Figure 2:**
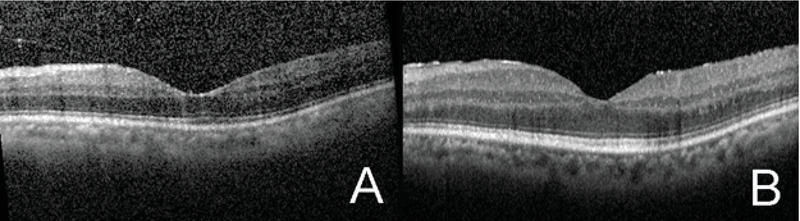
(A) OCT of the macula of the left eye before treatment initiation showing vitreomacular adhesion and few vitreous cells. (B) OCT of the macula of the left eye after 10 weeks from treatment initiation showing a fine epiretinal membrane.

**Figure 3:**
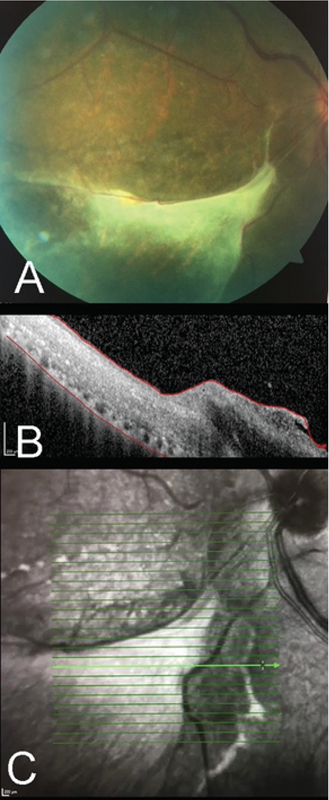
The inferonasal fundus photo of the left eye at 10 weeks showing preretinal fibrosis with a corresponding OCT (over the white lesion, see Arrow) showing an atrophic retina with resolved vitritis. A corresponding infrared image showing the line of scan of the OCT.

The patient was administered with 0.05 mL ganciclovir (4 mg/0.1 mL) and 0.10 mL foscarnet (2.4 mg/0.1 mL) intravitreal injections on diagnosis. Subsequently, his symptoms improved, and a 10-week course of oral valganciclovir (900 mg BID for 21 days followed by 900 mg QD for seven weeks) was administered. There was also a decrease in the dosage and eventual cessation of MMF with initiation of intravenous immunoglobulins. His lymphocytes improved to 9.8% (lymphocyte count, 0.7×10${}^{3}$ cells/μL; leukocyte count, 6.9×10${}^{3}$ cells/μL).

On valganciclovir discontinuation in week 10, the patient had a visual acuity of 20/25 OS with no inflammation and improvements in retinal hemorrhages and lesions. An epiretinal membrane was observed on macular optical coherence tomography (Figure 2). The inferonasal retina showed inactive whitish atrophy (Figure 3).

##  DISCUSSION

CMV retinitis is the most common ocular opportunistic infection associated with acquired immune deficiency syndrome.^[1]^ The prevalence of CMV retinitis in HIV patients has decreased since the advent of highly active antiretroviral therapy (HAART).^[2]^ However, the rate of CMV retinitis in non-HIV patients is increasing, likely due to the use of aggressive immunosuppressive agents.^[1]^ CMV is an infectious complication frequently associated with MMF.^[3]^

Visual prognosis of CMV infection in non-HIV patients is similar to that in HIV patients with poor visual outcomes associated with retinal detachments and macular involvement.^[1]^ CMV retinitis in patients with concomitant HIV infection lacks vitreous involvement.^[2]^ In contrast, vitritis is more commonly associated with non-HIV-related CMV retinitis infections.^[2]^ This is consistent with our patient's presentation.

CMV retinitis treatment in HIV patients involves HAART and antiviral therapy.^[1]^ In non-HIV patients, different etiologies of an immunocompromised state must be considered. Commonly used treatment strategies include systemic ganciclovir, foscarnet, valganciclovir, and intravitreal ganciclovir. Our patient received one initial intravitreal injection each of ganciclovir and foscarnet, as well as oral valganciclovir. Intravitreal injections are important for the treatment of vision-threatening CMV infections and were used in our case.^[4]^ Nevertheless, the mainstay treatment of CMV retinitis remains systemic antivirals, and it is not always necessary to start with intravitreal injections. The combination of intravitreal ganciclovir and foscarnet is effective in treating CMV retinitis.^[5]^

In summary, we presented a patient with MG who developed CMV retinitis due to immunosuppression as a result of MMF treatment. He was treated successfully with intravitreal and systemic antivirals.

##  Financial Support and Sponsorship

Nil.

##  Conflicts of Interest

There are no conflicts of interest.
